# Transfer deep learning and explainable AI framework for brain tumor and Alzheimer's detection across multiple datasets

**DOI:** 10.3389/fmed.2025.1618550

**Published:** 2025-06-19

**Authors:** Shtwai Alsubai, Stephen Ojo, Thomas I. Nathaniel, Mohamed Ayari, Jamel Baili, Ahmad Almadhor, Abdullah Al Hejaili

**Affiliations:** ^1^College of Computer Engineering and Sciences, Prince Sattam bin Abdulaziz University, Al-Kharj, Saudi Arabia; ^2^Department of Electrical and Computer Engineering, College of Engineering, Anderson University, Anderson, SC, United States; ^3^School of Medicine Greenville, University of South Carolina, Columbia, SC, United States; ^4^Faculty of Computing and Information Technology, Northern Border University, Arar, Saudi Arabia; ^5^Department of Computer Engineering, College of Computer Science, King Khalid University, Abha, Saudi Arabia; ^6^Department of Computer Engineering and Networks, College of Computer and Information Sciences, Jouf University, Sakaka, Saudi Arabia; ^7^Faculty of Computers and Information Technology, Computer Science Department, University of Tabuk, Tabuk, Saudi Arabia

**Keywords:** MRI image classification, transfer learning, explainable AI (XAI), hybrid CNN-VGG16 model, brain tumors, Alzheimer's disease, SHAP, medical imaging

## Abstract

**Introduction:**

The pressing need for accurate diagnostic tools in the medical field, particularly for diseases such as brain tumors and Alzheimer's, poses significant challenges to timely and effective treatment.

**Methods:**

This study presents a novel approach to MRI image classification by integrating transfer learning with Explainable AI (XAI) techniques. The proposed method utilizes a hybrid CNN-VGG16 model, which leverages pre-trained features from the VGG16 architecture to enhance classification performance across three distinct MRI datasets: brain tumor classification, Alzheimer's disease detection, and a third dataset of brain tumors. A comprehensive preprocessing pipeline ensures optimal input quality and variability, including image normalization, resizing, and data augmentation.

**Results:**

The model achieves accuracy rates of 94% on the brain tumor dataset, 81% on the augmented Alzheimer dataset, and 93% on the third dataset, underscoring its capability to differentiate various neurological conditions. Furthermore, the integration of SHapley Additive exPlanations (SHAP) provides a transparent view of the model's decision-making process, allowing clinicians to understand which regions of the MRI scans contribute to the classification outcomes.

**Discussion:**

This research demonstrates the potential of combining advanced deep learning techniques with explainability to improve diagnostic accuracy and trust in AI applications within healthcare.

## 1 Introduction

Brain tumors constitute a critical subset of central nervous system (CNS) disorders, with pathologies ranging from slow-growing benign masses to highly aggressive malignant neoplasms (1). Malignant types such as glioblastomas and anaplastic astrocytomas are particularly concerning due to their rapid proliferation, high invasiveness, and poor prognosis (2). The five-year relative survival rate for adults remains around 35.6%. These metastatic tumors are especially challenging due to their rapid infiltration into brain parenchyma and resistance to conventional therapies (3). The World Health Organization (WHO) classifies CNS tumors into grades I–IV based on histopathological, immunohistochemical, and molecular features (4), underscoring the need for early and accurate grading to guide clinical interventions.

Magnetic Resonance Imaging (MRI) remains the gold standard for brain tumor diagnosis and grading due to its superior soft tissue contrast and non-invasive nature (5). Advanced MRI modalities: such as T1-weighted (T1), contrast-enhanced T1 (T1C), T2-weighted (T2) (6), Fluid Attenuated Inversion Recovery (FLAIR) (7), Diffusion Tensor Imaging (DTI), Perfusion MRI, and MR Spectroscopy (MRS) (8) offer rich, multi-parametric information on tumor morphology, oedema, necrosis, vascularity, and infiltration (9). However, the manual interpretation of these high-dimensional images is time-consuming, prone to inter-observer variability, and particularly burdensome in resource-constrained settings with radiologist shortages (10). Tumor heterogeneity and overlapping imaging phenotypes further complicate diagnosis, prompting increased adoption of automated analysis tools powered by AI (11).

In parallel, neurodegenerative disorders like Alzheimer's disease (AD) pose unique diagnostic challenges. AD is characterized by progressive cognitive decline and structural brain changes such as cortical thinning and hippocampal atrophy, visible in MRI scans (12). Due to the limited availability of labeled data for early AD diagnosis, data augmentation techniques such as affine transformations, intensity scaling, noise injection, and GAN-based synthesis have been employed to improve model robustness (13). These enriched datasets also facilitate sequential transfer learning, enabling the repurposing of knowledge from AD-related imaging to other neurological domains, including brain tumor classification (14). Convolutional Neural Networks (CNNs) have tremendously succeeded in medical image classification, segmentation, and anomaly detection. Pre-trained architectures such as VGG16, ResNet, and DenseNet, initially developed for natural image datasets like ImageNet, can be fine-tuned via transfer learning to perform effectively in medical contexts (15).

This work proposes a novel hybrid framework that integrates a pre-trained VGG16 backbone with custom CNN layers and applies a sequential transfer learning strategy across three structurally distinct MRI datasets: a brain tumor, Alzheimer's disease, and an independent validation set. This approach leverages domain-relatedness in neuroimaging to enhance feature generalization and classification accuracy across multiple brain pathologies. Despite their high predictive performance, deep learning models are often criticized for their “black-box” nature, which limits interpretability and clinical trust (16). To overcome this limitation, we incorporate SHapley Additive exPlanations (SHAP), an explainable AI (XAI) method that attributes the model's output to specific pixels or regions in the input image. SHAP values offer visual insight into the regions most influential to model decisions, aligning them with anatomical structures and facilitating clinician interpretation. By striking a balance between high performance and interpretability, our framework presents a promising solution for real-world deployment in neuroimaging diagnostics.

This work proposes a novel hybrid framework that integrates a pre-trained VGG16 backbone with custom CNN layers and applies a sequential transfer learning strategy across three structurally distinct MRI datasets: a brain tumor, Alzheimer's disease, and an independent validation set. This approach leverages domain-relatedness in neuroimaging to enhance feature generalization and classification accuracy across multiple brain pathologies. Despite their high predictive performance, deep learning models are often criticized for their “black-box” nature, which limits interpretability and clinical trust (16). To overcome this limitation, we incorporate SHapley Additive exPlanations (SHAP), an explainable AI (XAI) method that attributes the model's output to specific pixels or regions in the input image. SHAP values offer visual insight into the regions most influential to model decisions, aligning them with anatomical structures and facilitating clinician interpretation. By striking a balance between high performance and interpretability, our framework presents a promising solution for real-world deployment in neuroimaging diagnostics.

The proposed method begins with preprocessing all datasets, including normalization, resizing, augmentation, and partitioning into train/validation/test splits. A hybrid CNN architecture is then constructed by combining frozen VGG16 features with custom convolutional and dense layers. The model is trained on a brain tumor dataset and then fine-tuned sequentially on an Alzheimer's dataset and a third validation dataset using transfer learning. Each stage involves model reconfiguration and controlled unfreezing of layers. Finally, SHAP-based explainability is applied to visualize model decisions, and performance is evaluated using standard metrics such as accuracy, precision, recall, F1-score, and confusion matrices.

**Figure 1:**
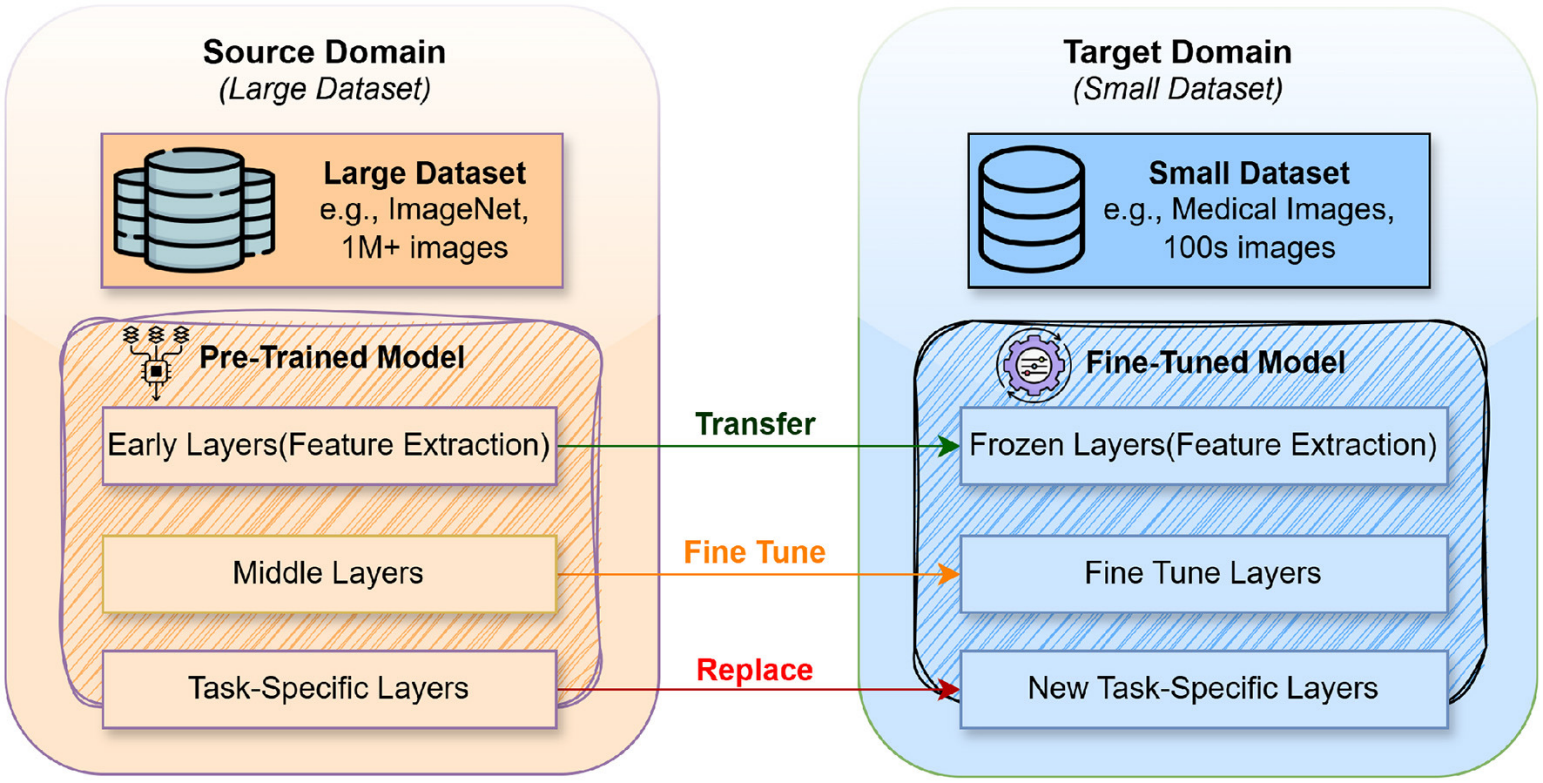
How transfer learning works.

Figure 1 illustrates the concept of transfer learning, a technique in machine learning where knowledge gained from a source domain is utilized to enhance learning in a target domain. The source domain comprises a large dataset, such as ImageNet, which contains over a million images. A pre-trained model is developed using this extensive dataset, comprising three key components: early layers for feature extraction, middle layers, and task-specific layers. In transfer learning, the early layers that capture general features like edges and textures are transferred to the model for the target domain, where data is limited, such as a medical image dataset with only hundreds of samples. These layers become “frozen” in the fine-tuned model, meaning they are not updated during training on the small dataset. The middle layers are fine-tuned, and the adjustments are based on the new data to capture domain-specific features better. Finally, the task-specific layers from the source model are replaced with new ones tailored to the target domain's specific task.

### 1.1 Research contributions

The major research contributions of this study are the following:

- A novel approach that leverages a pre-trained VGG16 model combined with custom CNN layers, using sequential transfer learning across three distinct MRI datasets (brain tumor, Alzheimer's, and validation) to improve classification accuracy while requiring minimal training data.
- This study demonstrates effective knowledge transfer between different neurological conditions (from brain tumor classification to Alzheimer's detection), showing that features learned from one medical imaging domain can enhance performance in related but distinct diagnostic tasks. A comprehensive preprocessing pipeline, including image normalization, resizing, and data augmentation, is implemented to improve model robustness and generalizability across datasets with varying characteristics.
- This research incorporates SHapley Additive exPlanations (SHAP) analysis to provide transparent, pixel-level attribution of model decisions, addressing the “black box” problem of deep learning in healthcare by enabling clinicians to understand which regions of MRI scans influence diagnostic classifications.

### 1.2 Research organization

This research is organized into the following main sections. Section 2 presents related work, discussing recent advances in deep learning for medical imaging, the effectiveness of transfer learning, and the growing importance of XAI in healthcare. Section 3 outlines the proposed framework, detailing integrating pre-trained convolutional neural networks with XAI methods, such as Grad-CAM, to enhance performance and interpretability. Section 4 presents the experimental analysis, which includes dataset description, evaluation metrics, and results comparing the proposed model with existing techniques. Finally, Section 5 concludes the study by summarizing key findings and suggesting directions for future research.

## 2 Related work

This section presents related work, discussing recent advances in deep learning for medical imaging, the effectiveness of transfer learning, and the growing importance of XAI in healthcare. Tuncer et al. (17) proposed a lightweight convolutional neural network named FiboNeXt for Alzheimer's disease classification using MRI images. The model was designed by integrating ConvNeXt architecture elements, attention, and concatenation layers. The dataset was divided into four classes and included both original and augmented versions, where the augmented data was used for training and the original for testing. The primary aim was to achieve high accuracy with fewer trainable parameters. Experimental results demonstrated that FiboNeXt achieved 95.40 and 95.93% validation accuracy on two datasets, while test accuracy reached 99.66 and 99.63%, respectively, highlighting the model's efficiency and generalization capability. An optimized hybrid transfer learning (TL) framework was introduced by Lasagni et al. (18) to classify brain tumors using MRI images. The approach combined advanced preprocessing techniques, such as noise reduction and contrast enhancement, with an ensemble of pretrained deep learning models, VGG16 and ResNet152V2. The framework achieved an impressive classification accuracy of 99.47% on a complex four-class dataset. Explainable AI (XAI) methods like SHAP and Grad-CAM were employed to ensure transparency and clinical trust. These tools provided visual and quantitative insights into model predictions, facilitating better interpretability and making the model more suitable for real-world clinical applications.

Bhaskaran and Datta (19) investigated the use of 3D convolutional neural networks (3D-CNNs) for detecting focal cortical dysplasia (FCD) from a dataset containing MRI scans of 170 individuals (85 patients and 85 controls). They studied the advantages of cross-modality transfer learning using pretrained ResNet variants (ResNet-18, -34, and -50, trained initially on segmentation tasks). Transfer learning significantly improved classification performance to up to 80.3%. Moreover, they also introduced a novel Heat-Score, a combination of Grad-CAM, to evaluate the model interpretability. The model was able to fill the gap between AI predictions and expert diagnostic insights by using this metric, showing the model's effectiveness in identifying clinically relevant seizure zones. Tonni et al. (20) used the InceptionV3 architecture to classify brain MRI images into three tumor types (meningioma, glioma and pituitary) with different embeddings initialization for imagenet and the studied data. Several open-source XAI tools were integrated to address the challenge of model interpretability, including LIME, SHAP, and Grad-CAM. The model attained a classification accuracy of 93% and an F1-score of 0.93. Among the XAI tools, SHAP provided the highest level of explainability at ~60%, aligning better with expert-identified tumor regions. In contrast, LIME and Grad-CAM explained < 50% of the cases. The findings revealed that non-tumor-related features had a notable impact on model predictions, suggesting a need for further refinement in feature attribution techniques.

Nahiduzzaman et al. (21) proposed a novel framework that integrates a lightweight parallel depthwise separable convolutional neural network (PDSCNN) with a hybrid ridge regression extreme learning machine (RRELM) for classifying four brain tumor types (glioma, meningioma, pituitary, and no tumor) using MRI images. The approach utilizes contrast-limited adaptive histogram equalization (CLAHE) to enhance tumor feature visibility, followed by PDSCNN for efficient tumor-specific feature extraction with reduced computational cost. To improve classification performance, a ridge regression-enhanced ELM (RRELM) is introduced, addressing the limitations of traditional ELMs. Comparative analysis with state-of-the-art models revealed that the proposed PDSCNN-RRELM achieved superior results, with average precision, recall, and accuracy reaching 99.35%, 99.30%, and 99.22% through five-fold cross-validation. Vanaja et al. (22) proposed a diagnostic framework for Alzheimer's Disease (AD) by leveraging machine learning and a customized deep convolutional neural network (cDCNN) with three convolutional layers applied to MRI data. The analysis incorporates two datasets, Alzheimer's Disease Neuroimaging Initiative (ADNI) and a Kaggle dataset, to examine diverse subject groups and imaging characteristics linked to AD pathology. To mitigate class imbalance, the Synthetic Minority Over-sampling Technique (SMOTE) is employed. Traditional machine learning classifiers such as support vector machine, k-nearest neighbor, random forest, decision trees, and XGBoost are evaluated alongside the cDCNN model, which focuses on key MRI biomarkers of AD. The cDCNN achieved 87% accuracy on the ADNI dataset despite preprocessing challenges due to converting DICOM images to JPEG, which affected image quality.

Joshi et al. (23) introduced a transfer learning approach for classifying Parkinson's disease using the imbalanced PPMI dataset, leveraging Big Transfer (BiT) models. These pre-trained models utilize Group Normalization with Weight Standardization and adopt BiT-HyperRule for effective fine-tuning across diverse datasets. Various BiT architectures, including BiT-S and BiT-M variants, were evaluated. The best-performing model, BiT-M152x4, achieved 86.71% accuracy, surpassing the previous state-of-the-art RA-GCN model (76%). Additionally, the same BiT models were applied to the imbalanced BCCD dataset, where BiT-M152x4 again outperformed VGG16 (98.52% vs. 74%), demonstrating the versatility and robustness of the proposed approach. Bin Shabbir Mugdha and Uddin (24) conducted a comparative analysis between a newly developed Convolutional Neural Network (CNN) model and several pre-trained models using transfer learning, including VGG-16, ResNet-50, AlexNet, and Inception-v3. VGG-16 achieved the best performance among all models with a test accuracy of 95.52%, training accuracy of 99.87%, and a validation loss of 0.2348. ResNet-50 followed with 93.31% test accuracy, 98.78% training accuracy, and 0.6327 validation loss. The custom CNN model achieved 92.59% test accuracy, 98.11% training accuracy, and a validation loss of 0.2960. Inception-v3 showed the lowest performance with 89.40% test accuracy and a validation loss of 0.4418.

Khedgaonkar et al. (25) proposed a Graph Neural Network (GNN)-based approach for brain MRI classification, addressing the limitations of traditional methods in integrating spatial and frequency domain features. By applying Fourier, Gabor, and convolutional transformations, key features are extracted and fused into a unified representation. MRI images are modeled as nodes in a graph, capturing structural and semantic relationships. The GNN leverages this graph structure to learn discriminative features through neighborhood aggregation. The method demonstrated superior performance across precision, accuracy, recall, specificity, AUC, and delay, outperforming conventional techniques. Ilani et al. (26) focused on classifying brain tumors glioma, meningioma, and pituitary using MRI scans, leveraging the U-Net architecture for segmentation alongside transfer learning-based CNN models such as Inception-V3, EfficientNetB4, and VGG19. Model performance was evaluated using F-score, recall, precision, and accuracy metrics. U-Net outperformed other models, achieving 98.56% accuracy, a 99% F-score, 99.8% AUC, and 99% recall and precision. It also maintained strong generalization with 96.01% accuracy in cross-dataset validation using an external cohort. The results highlight U-Net's effectiveness in precise brain tumor segmentation, supporting early diagnosis and treatment planning.

Rasool et al. (27) proposed ResMHA-Net, a deep learning framework combining ResNet residual blocks with multi-head attention to enhance glioma segmentation in 3D MRI. This architecture captured long-range dependencies and emphasized informative regions, improving the segmentation of complex glioma sub-regions. It was trained and validated on BraTS 2018–2021 datasets, with the best performance observed on BraTS 2021, demonstrating strong adaptability. Predicted masks from three datasets were used to extract radiomic features, which, along with clinical data, trained an ensemble model for survival prediction. This model employed a voting mechanism across multiple learners and achieved a 73% overall survival prediction accuracy. Gasmi et al. (28) developed an ensemble classification model integrating Vision Transformers (ViT) and EfficientNet-V2 to capture both global and local features from brain MRI. Model outputs were combined using a genetic algorithm-optimized weighted ensemble, which selected the best combination to maximize accuracy. Trained on a labeled MRI dataset, the ensemble model outperformed individual and traditional classifiers, achieving a 95% classification accuracy with improved precision, recall, and F1-score.

While these studies have achieved high accuracy through various architectures and optimization techniques, many face limitations such as reliance on single-domain datasets, limited transferability across neurological disorders, or insufficient interpretability. Most existing works focus on enhancing performance or providing visual explanations, but few offer a unified framework that balances generalization, accuracy, and explainability across diverse brain pathologies. Furthermore, many methods lack rigorous evaluation of independent datasets, raising concerns about overfitting and real-world applicability. Our work addresses these gaps by proposing a multi-stage transfer learning strategy that spans distinct MRI datasets and integrating SHAP for transparent, clinically meaningful explanations.

## 3 Proposed framework

**Figure 2:**
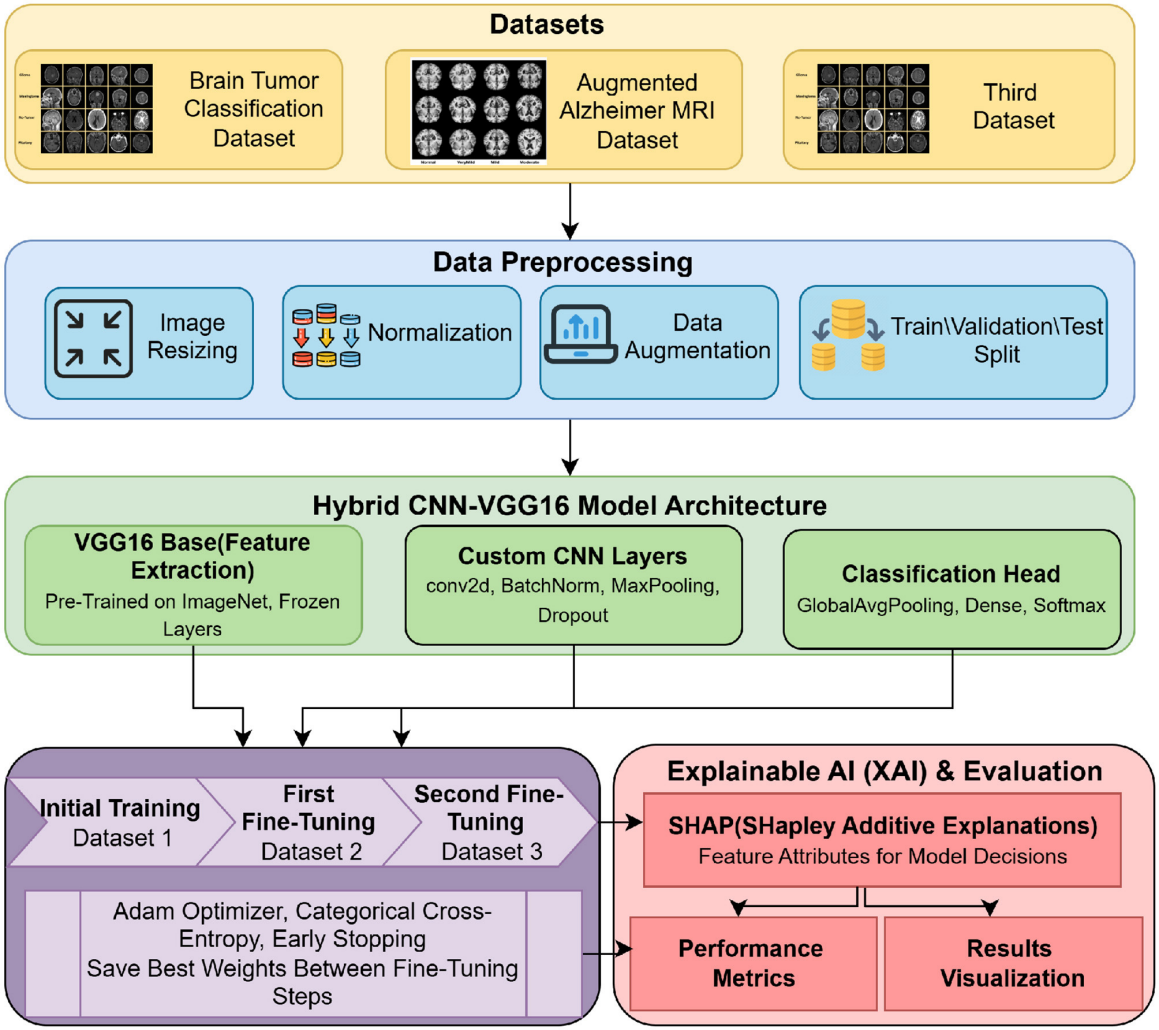
Hybrid CNN-VGG16 model with transfer learning and XAI for MRI classification.

This section explains the proposed framework, detailing the integration of pre-trained convolutional neural networks with XAI methods like Grad-CAM to improve performance and interpretability. The workflow of the proposed framework is illustrated in Figure 2. The figure presents a comprehensive pipeline for a Hybrid CNN-VGG16 model designed for MRI image classification, which leverages transfer learning and explainable artificial intelligence (XAI) techniques. The process is divided into five primary stages: datasets, data preprocessing, model architecture, training, and evaluation with XAI. The first stage highlights the use of three distinct datasets: the Brain Tumor Classification Dataset (with classes like glioma, meningioma, no tumor, and pituitary), the Augmented Alzheimer MRI Dataset (including mild, moderate, non-demented, and very mild demented classes), and a third dataset which again covers brain tumor categories. These datasets undergo different preprocessing steps, such as image resizing, normalization, augmentation, and dataset splitting into training, validation, and testing sets. Next, the Hybrid CNN-VGG16 model architecture is detailed. It begins with the VGG16 base model pretrained on ImageNet with frozen layers used for feature extraction. On top of this base, custom convolutional layers (including Conv2D, batch normalization, max pooling, and dropout) are added to enhance learning. The final part of the model is the classification head, which includes global average pooling, dense layers, and a softmax layer for multi-class output. The training process is conducted in three sequential phases. It starts with initial training on the brain tumor dataset, followed by two fine-tuning stages on the Alzheimer dataset and then on the third dataset. The training uses the Adam optimizer, categorical cross-entropy loss, and early stopping, with the best-performing model weights preserved between each stage. Finally, the Explainable AI (XAI) & Evaluation block involves model interpretation and performance assessment. SHapley Additive exPlanations (SHAP) provides feature attributions, allowing insight into how the model makes decisions. Additionally, several performance metrics such as accuracy, F1-score, precision, and recall are used, and visual results are presented via confusion matrices and SHAP plots.

Algorithm 1 defines a general process to adapt a pre-trained source model *M*_*S*_ to a new target task using the target dataset *D*_*T*_. The source model is cloned to create the target model *M*_*T*_, after which selected layers are frozen based on the strategy ϕ. The final output layer is replaced to align with the target labels, and the dataset *D*_*T*_ is split into training, validation, and test subsets. Fine-tuning is performed over *E* epochs using gradient descent on trainable parameters, with early stopping optionally applied. The algorithm also supports the progressive unfreezing of layers for staged fine-tuning. The final model is evaluated on the $D_{T}^{test}$ test set. Specifically, the following terms are: *M*_*S*_ denotes the pre-trained source model, and *M*_*T*_ is the target model initialized as a clone of *M*_*S*_. The target dataset is represented as $D_{T}={\left\{\left(x_{i},y_{i}\right)\right\}}_{i=1}^{N_{T}}$, where *x*_*i*_ is an input sample, *y*_*i*_ is the corresponding target label, and *N*_*T*_ is the total number of samples. The learning rate is denoted by α, and *E* represents the number of training epochs. The strategy ϕ defines which layers in *M*_*T*_ will be frozen or trainable during fine-tuning. Each mini-batch is represented by $B={\left\{\left(x_{j},y_{j}\right)\right\}}_{j=1}^{b}$, where *b* is the batch size. For each sample *x*_*j*_ in the batch, ŷ_*j*_ is the predicted output by *M*_*T*_. The loss for a batch is computed as $L=\frac{1}{b}\underset{j}{\sum }\ell \left({ŷ}_{j},y_{j}\right)$, where ℓ is a loss function such as cross-entropy. The model parameters are denoted by θ, and gradient descent updates them via $\theta \leftarrow \theta -\alpha \nabla_{\theta }L$. The dataset *D*_*T*_ is split into training, validation, and test sets, denoted by $D_{T}^{train}$, $D_{T}^{val}$, and $D_{T}^{test}$, respectively. Additionally, if progressive unfreezing is enabled, layers are incrementally unfrozen in *S* stages, with each stage using its learning rate α_*s*_ and epoch count *E*_*s*_.

**Algorithm 1:**
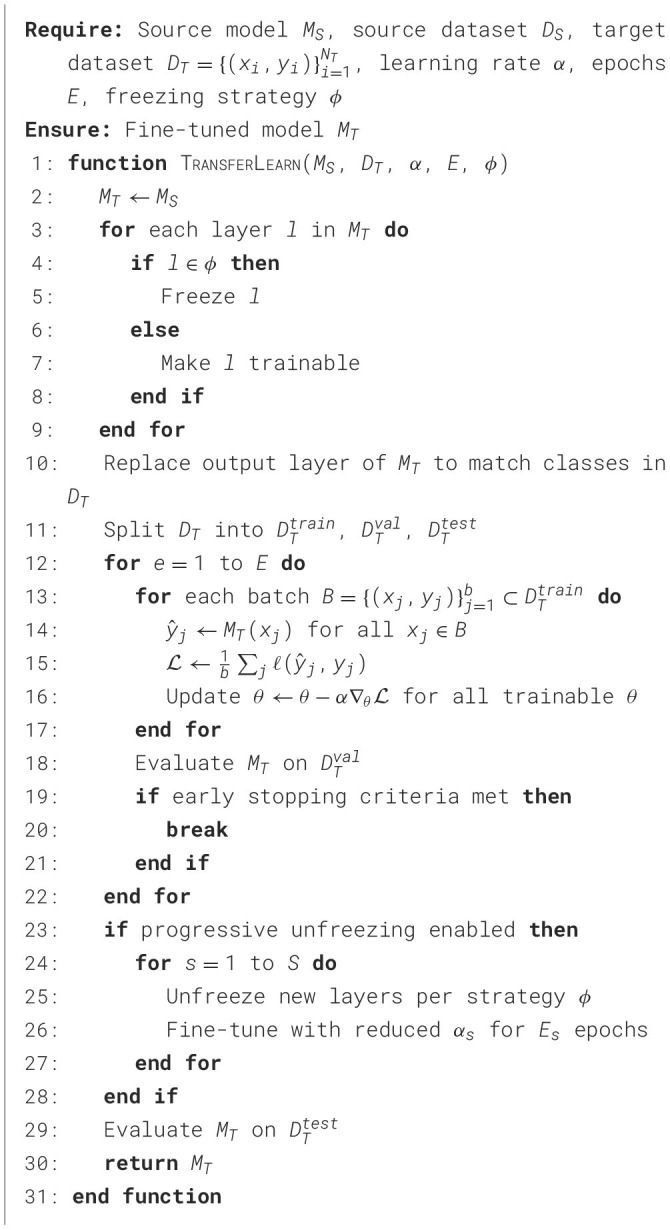
Transfer learning for neural network models.

Algorithm 2 details a pipeline for MRI image classification using three datasets. The datasets are defined as follows: $D_{1}={\left\{\left(x_{i}^{1},y_{i}^{1}\right)\right\}}_{i=1}^{N_{1}}$ corresponds to the Brain Tumor Dataset (BTD), $D_{2}={\left\{\left(x_{i}^{2},y_{i}^{2}\right)\right\}}_{i=1}^{N_{2}}$ is the Alzheimer Dataset (AD), and $D_{3}={\left\{\left(x_{i}^{3},y_{i}^{3}\right)\right\}}_{i=1}^{N_{3}}$ is the third validation dataset (VD). Here, $x_{i}^{k}$ is an MRI image, and $y_{i}^{k}$ is its corresponding label for dataset *D*_*k*_ with *N*_*k*_ samples. The learning rate, batch size, and number of epochs for training on dataset *D*_*k*_ are represented by α_*k*_, *B*_*k*_, and *E*_*k*_, respectively. During preprocessing, each image *x*_*i*_ is normalized by subtracting the mean μ and dividing by the standard deviation σ, then resized to a fixed height *h* and width *w*. Augmentation is applied through transformation functions *T*(*x*_*i*_), and the dataset is split into training, validation, and test subsets. The model is constructed using a pretrained VGG16 backbone denoted as *V*, from which features *F* are extracted. These features are frozen and connected to additional convolutional, batch normalization (BN), max pooling, dropout, global average pooling (GAP), and dense layers, ending with a final dense output layer with *C* units representing the number of classes. The function Train compiles the model with the Adam optimizer (learning rate α) and categorical cross-entropy (CCE) loss, then fits it on the training set and evaluates it on the test set. The function FineTune replaces the output head with *C* classes, unfreezes the last layers for fine-tuning, recompiles the model, and continues training. The Explain function employs DeepExplainer from SHAP to generate saliency maps *S*_*i*_ for test samples *x*_*i*_, where *X*_*bg*_ is a background dataset used for explanations. The predicted label for a sample is given by ŷ_*i*_ = argmax*M*(*x*_*i*_). The evaluation function computes standard metrics: accuracy (Acc), F1-score (F1), precision (Prec), recall (Rec), and confusion matrices. These changes have been incorporated to improve the transparency of the algorithm.

**Algorithm 2:**
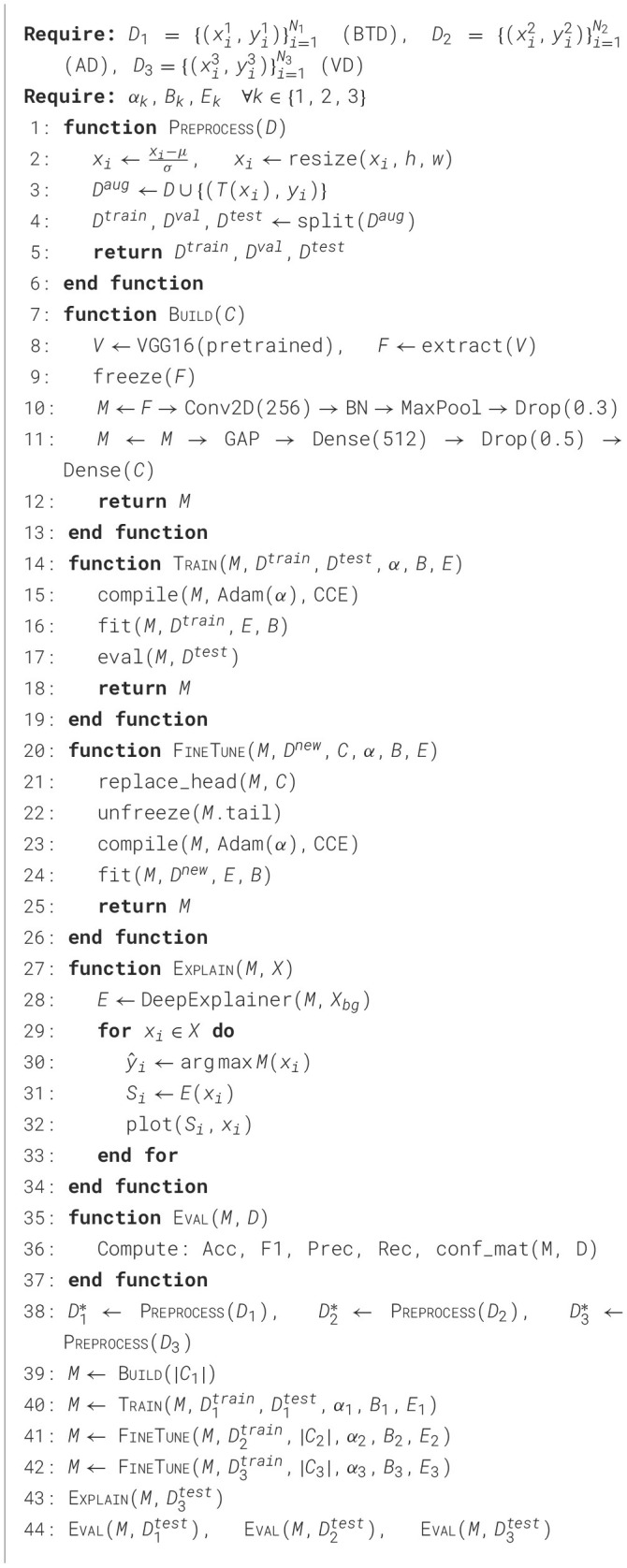
Hybrid CNN-VGG16 with TL and XAI for MRI classification.

### 3.1 Experimental dataset

In this research, we utilized three datasets for classifying MRI images by training deep learning models. The first dataset (https://www.kaggle.com/datasets/sartajbhuvaji/brain-tumorclassification-mri, accessed March 25, 2025) is based on Brain tumors, among the most aggressive diseases affecting children and adults, comprising 85%–90% of all primary Central Nervous System (CNS) tumors. Annually, ~11,700 new brain tumor cases are reported, with a 5-year survival rate of 34% for men and 36% for women. Tumors are categorized into the following types: Glioma Tumor, Meningioma Tumor, No Tumor, and Pituitary Tumor. The second dataset (https://www.kaggle.com/datasets/uraninjo/augmented-alzheimer-mri-dataset, accessed March 25, 2025) used in this research is the Augmented Alzheimer's MRI Dataset. It contains brain MRI images classified into four categories: Non-Demented, Very Mild Demented, Mild Demented, and Moderate Demented. The dataset is organized into two main folders, one containing the original images and the other containing augmented versions to increase data variability. Both training and testing sets include samples from all four classes. Augmented data helps improve deep learning models' performance and generalization capability in classifying different stages of Alzheimer's disease. The third dataset (https://www.kaggle.com/datasets/sartajbhuvaji/brain-tumor-classification-mri, accessed March 25, 2025) used in this research is a combined brain tumor MRI dataset derived from three sources: Figshare, the SARTAJ dataset, and the Br35H dataset. It contains 7,023 MRI images classified into four categories: Glioma, Meningioma, Pituitary, and No Tumor. Images for the “No Tumor” class were taken from the Br35H dataset. Due to misclassification issues observed in the Glioma class of the SARTAJ dataset, which was identified through inconsistent model performance and validation against other research, those images were removed and replaced with correctly labeled images from the Figshare dataset. This curated dataset supports the classification of brain tumors, which can be either benign or malignant and is critical for early diagnosis, given the life-threatening nature of tumor-induced pressure within the skull.

### 3.2 Data preprocessing

The preprocessing process begins with loading each MRI image and converting it from the default Blue-Green-Red (BGR) color format to the standard Red-Green-Blue (RGB) format to ensure compatibility with deep learning models. This conversion maintains consistency in color representation across all images, preventing misinterpretation of visual features during training and improving the accuracy of tumor classification. After converting the image to RGB, the next preprocessing step involves resizing each image to a fixed dimension of 128 × 128 pixels. Neural networks require input data to have a consistent shape, and resizing ensures that all images, regardless of their original resolution, meet the input requirements of the model. Specifically, resizing transforms an image *IϵR*^*H*×*W*×3^ into a standardized format *I*′ϵ*R*^128 × 128 × 3^, where *H* and *W* represent the original height and width of the image, respectively. This step ensures that all input images are of uniform size, allowing for efficient model training and processing. After resizing, the pixel values of the images are normalized by scaling them from the original range of [0, 255] to [0, 1]. This is achieved by dividing each pixel value by 255 (see Equation 1):


(1) $$\begin{array}{l} I_{norm}=\frac{I}{255} \end{array}$$

Normalization helps stabilize and accelerate the neural network's learning process by ensuring the input data has a smaller, more uniform range of values. It also helps reduce the internal covariate shift, thus enabling more effective weight updates during training.

The class labels, initially string values such as “glioma_tumor,” “meningioma_tumor,” etc., are converted into a numerical format using a label map. Each label is then one-hot encoded using the to_categorical() function. One-hot encoding transforms categorical labels into a binary matrix where only the index of the class is marked as 1, and all others are 0. For instance, the label “glioma_tumor” becomes [1, 0, 0, 0]. This format is compatible with multi-class classification models. In mathematical terms, for a class *C*∈{0, 1, 2, 3}, the one-hot encoded vector *y* is defined as shown in Equation 2:


(2) $$y_{i}=\{\begin{array}{ll} 1, & \text{if }i=C\\ 0, & \text{otherwise} \end{array}\text{ for }i\in \{0,1,2,3\}$$

It is essential to evaluate model performance and prevent overfitting; therefore, this study utilized train_test_split() to divide the dataset into training and validation sets. Specifically, 80% of the data was allocated for training and 20% for validation. The use of a fixed random_state ensured reproducibility. This separation allowed the model to be assessed on unseen data, providing a more precise measure of its generalization capability.

### 3.3 Data augmentation

The model generalization should be improved together with mitigating overfitting if we have a small dataset. For this, this study expanded the training data using data augmentation techniques, which artificially increased the training dataset by generating simple variations of the images. These variations make the model more robust and well-performing for real-world transformations that may occur in medical imaging.

The operations applied in this study for augmentation are random rotations in a ±20-degree range, horizontal and vertical translations of 20% of the Image dimensions, shear transformation with moderate intensity, zooming in ± 20% and random horizontal flips to simulate different orientations. These transformations were chosen carefully to resemble variations in MRI scans that occur naturally and do not modify the underlying anatomical structures. The augmentation process can be formally described as applying a transformation function *T* to an input image *x*, resulting in an augmented image *x*′ (see Equation 3):


(3) $$\begin{array}{l} x^{\prime }=T\left(x\right) \end{array}$$

Where the transformation function *T* is a composition of individual operations, such as (see Equation 4):


(4) $$\begin{array}{l} \begin{array}{cc} x^{\prime }=R_{\theta }\left(x\right)\; & \text{(Rotation)}\\ x^{\prime }=T_{dx,dy}\left(x\right)\; & \text{(Translation)}\\ x^{\prime }=S_{\alpha }\left(x\right)\; & \text{(Shear)}\\ x^{\prime }=Z_{s}\left(x\right)\; & \text{(Zoom)}\\ x^{\prime }=F\left(x\right)\; & \text{(Flip)} \end{array} \end{array}$$

These operations ensure that the data is represented in diverse ways during the training process, thus increasing the chances for it to generalize better to unseen inputs. The augmentation parameters were fit to the training dataset before training, and these fit parameters were used in the training process. Hence, the behavior of transformation is consistent during the time of learning.

### 3.4 Model architecture

The details of the model architecture of CNN, Custom CNN, VGG16, ResNet and Hybrid CNN-VGG16 are discussed in this section.

#### 3.4.1 CNN model

The first model architecture specifically for the MRI image classification is the Convolutional Neural Network (CNN) (29). It takes input images of size 128 × 128 × 3 and starts with a Conv2D layer of 32 filters (3 × 3 kernel, ReLU) and, as usual, MaxPooling2D (2 × 2) to reduce spatial dimensions. It is followed by a Conv2D layer with 64 filters (3 × 3, ReLU) and another MaxPooling2D (2 × 2). Then, a third Conv2D layer with 128 filters (3 × 3, ReLU) and another MaxPooling2D layer (2 × 2) is added. It is then flattened and passed through a Dense layer with 128 neurons (ReLU) and a Dropout layer of 0.5 dropout rate to prevent overfitting. The Dense output layer with a softmax activation is used to classify the input into one of four classes: glioma, meningioma, no tumor and pituitary tumor. Lastly, we compile the model using the Adam optimizer and the categorical cross-entropy loss, which fit the multi-class classification correctly.

#### 3.4.2 Custom CNN model

The custom CNN model shares the core structure of the basic CNN three convolutional layers followed by max-pooling, flattening, a dense layer, dropout, and a softmax output for multi-class classification. However, it enhances the architecture by integrating Batch Normalization after each convolutional layer. This addition helps stabilize learning, speeds up convergence, and improves generalization. While the layer progression and classification targets remain the same, the inclusion of batch normalization distinguishes this model by offering better training dynamics and potentially higher performance (30).

#### 3.4.3 VGG16

The third model utilizes VGG16, a well-known deep CNN architecture pre-trained on the ImageNet dataset, as a feature extractor (31). Unlike the previous custom models, VGG16's convolutional layers are frozen to retain learned features, reducing training time and preventing overfitting on small datasets. On top of the frozen base, custom classification layers are added: a global average pooling layer to reduce feature maps, a dense layer with ReLU activation, a dropout layer for regularization, and a softmax output layer to classify MRI images into four tumor categories. This transfer learning approach combines the power of a proven model with task-specific tuning for improved accuracy and generalization.

#### 3.4.4 ResNeT

The fourth is a ResNet model that integrates residual connections for more efficient learning, especially in deeper networks (32). It starts with a convolutional layer followed by max-pooling, similar to previous models. The main distinction in this model is the use of residual blocks, which include two convolutional layers per block. The shortcut connections are added to the output of these blocks, enabling the model to bypass specific layers and help mitigate the vanishing gradient issue. In the second block, a 1 × 1 convolution is used to match the output dimensions of the shortcut. The rest of the architecture follows the same structure, with global average pooling, a dense layer, and a softmax output for classification. The model is optimized using Adam with a learning rate of 0.0001 and uses categorical cross-entropy for loss.

#### 3.4.5 Hybrid VGG16-CNN

The Hybrid CNN + VGG16 model integrates a pre-trained VGG16 model for feature extraction with a custom CNN designed to learn additional task-specific features (33). The VGG16 model, with its convolutional layers frozen, leverages the pre-learned features from the ImageNet dataset without any further updates during training. A Global Average Pooling layer processes its output to create a more compact representation of the features. The custom CNN learns additional features directly relevant to tumor classification. This CNN includes several convolutional layers followed by max-pooling layers to reduce the spatial dimensions of the feature maps. The resulting output is flattened and passed through a fully connected layer, with ReLU activation and a dropout layer for regularization. The features from both models are merged using the concatenate operation, followed by another fully connected layer with ReLU activation and a dropout layer. The final output layer uses softmax activation to produce a probability distribution over the four tumor categories: glioma tumor, meningioma tumor, no tumor, and pituitary tumor. The model is compiled with the Adam optimizer and categorical cross-entropy as the loss function, which is suitable for multi-class classification. It is trained for 50 epochs with a batch size of 32, using training and validation data.

### 3.5 Fine tuning models

The previously trained Hybrid CNN + VGG16 model was fine-tuned for the second experimentation phase using the Augmented Alzheimer's MRI dataset. This dataset includes four categories: Mild Demented, Moderate Demented, Non Demented, and Very Mild Demented. The hybrid model combines the VGG16 architecture, which was pre-trained on the ImageNet dataset and used as a frozen feature extractor, with a custom CNN trained to extract domain-specific features. To adapt the model for this new classification task, the final dense layer was replaced to match the four output classes. While the VGG16 layers remained frozen to retain their generalized feature representations, the custom CNN layers were set as trainable to learn patterns specific to Alzheimer's stages. Additionally, dropout and L2 regularization were applied to mitigate overfitting. The model was compiled using the Adam optimizer with a learning rate of 0.0005 and trained using augmented image data. To further validate our hybrid CNN+VGG16 model, we evaluated its performance on a third publicly available MRI brain tumor dataset consisting of four categories: glioma, meningioma, pituitary, and no tumor. The model architecture and training methodology remained consistent with previous experiments, incorporating dual-input feature fusion and transfer learning. After minor data augmentation and preprocessing adjustments, the model was retrained using a two-input pipeline and evaluated on stratified splits. The model demonstrated strong generalization to this new dataset, maintaining high accuracy across all classes. These results further reinforce the robustness and adaptability of our proposed hybrid model to varying data distributions.

To evaluate the generalization performance of the proposed Hybrid CNN + VGG16 model without relying on data augmentation, we conducted additional experiments on the unaltered original Alzheimer's MRI dataset. While the model architecture and configuration remained consistent, the training set consisted solely of original images, with no synthetic augmentation applied. The output layer was modified to match the four-class structure of this dataset. Only the custom CNN layers were updated during fine-tuning, while the VGG16 backbone remained frozen. The training used the same optimizer (Adam) and loss function (categorical cross-entropy) as in the augmented experiments. This experiment provides insight into how well the model performs in a more constrained, real-world scenario.

## 4 Experimental analysis and results

In this section, the accuracy, precision, recall, and F1 scores are used to assess the performance of the models. More specifically, it describes systematic experimental outcomes. This subsection defines all performance measurements, such as accuracy, precision, recall, and F1-score and indicates how these measurements must be used.

The number of correctly classified instances (TP + TN) is the total number of instances of the data set. By applying Equation 5, we can calculate this value:


(5) $$\begin{array}{l} Accuracy=\frac{TP+TN}{TP+FP+TN+FN} \end{array}$$

It is the ratio of the number of times the model accurately predicted a product to the total number of times it has predicted it positively. Applying Equation 6 in this way will provide this result:


(6) $$\begin{array}{l} Precision=\frac{TP}{TP+FP} \end{array}$$

The ratio of positive predictions to the data's actual number of positive instances. It reflects the model's ability to capture all positive instances. Use Equation 7 in the following manner to find this value:


(7) $$\begin{array}{l} Recall=\frac{TP}{TP+FN} \end{array}$$

The harmonic mean of precision and recall provides a single metric to balance both. It is beneficial when an imbalance between classes is calculated using Equation 8.


(8) $$\begin{array}{l} F1-score=2\times \frac{Precision+Recall}{Precision+Recall} \end{array}$$

**Figure 3:**
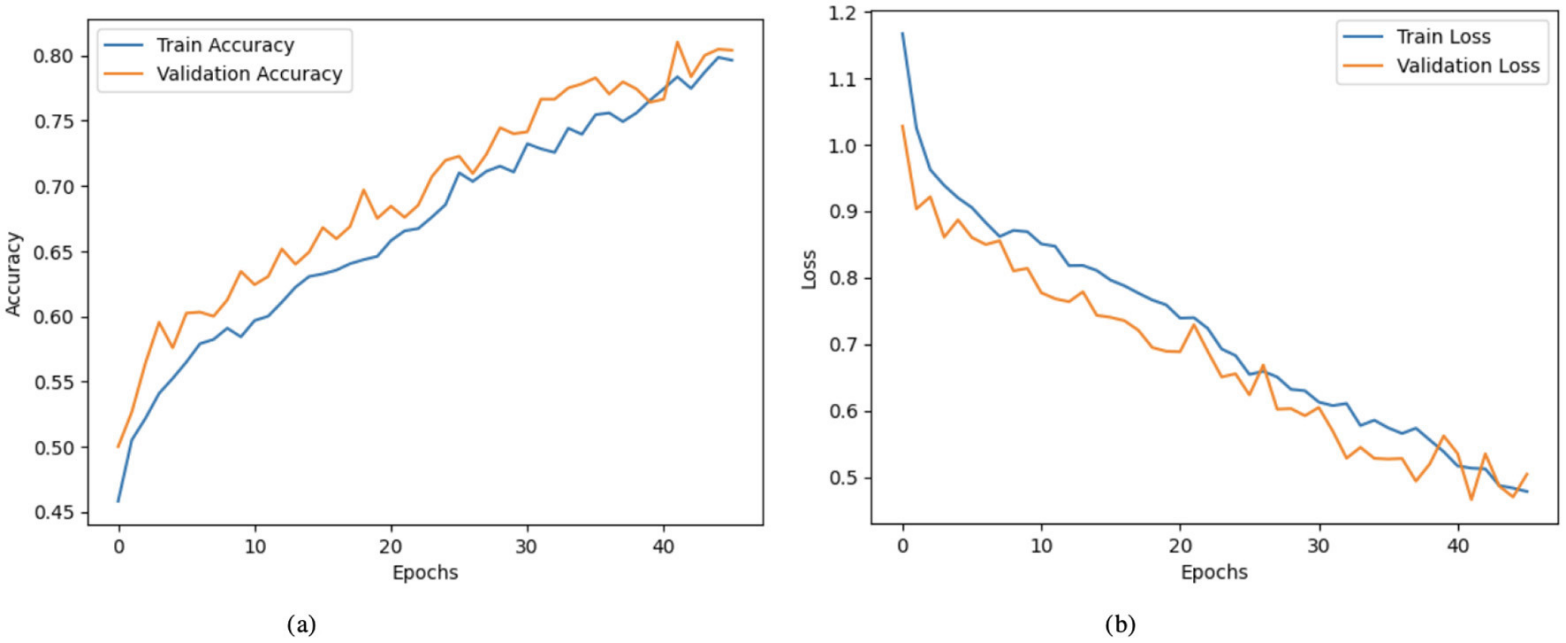
Graphical representation of hybrid CNN-VGG16 model with XAI on second dataset. **(a)** Accuracy graph. **(b)** Loss graph.

Figure 3a illustrates a model's training and validation accuracy over 45 epochs. The training accuracy commences at ~0.460 at the 0_*th*_ epoch and shows a steady upward trajectory, reaching about 0.800 by the 40_*th*_ epoch. Similarly, the validation accuracy begins at around 0.500 and follows a comparable increasing trend, surpassing the training accuracy at several points and culminating at ~0.805 at the final epoch. Figure 3b presents the corresponding loss values for training and validation over the same number of epochs. The training loss starts at around 1.17 at the 0_*th*_ epoch and declines progressively, reaching about 0.47 by the 40_*th*_ epoch. The validation loss follows a similar pattern, beginning near 1.02 and steadily decreasing to ~0.50 at the final epoch.

**Figure 4:**
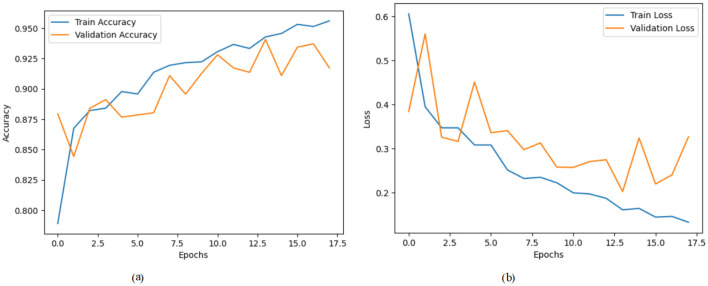
Graphical representation of hybrid CNN-VGG16 model with transfer learning on third dataset. **(a)** Accuracy graph. **(b)** Loss graph.

Figure 4a illustrates a model's training and validation accuracy over 17 epochs. The training accuracy begins at ~0.790 at the 0_*th*_ epoch and exhibits a consistent upward trend, reaching about 0.955 by the 17_*th*_ epoch. The validation accuracy initiates at around 0.880 and fluctuates slightly throughout the training process, peaking around the 14_*th*_ epoch near 0.935 before ending at ~0.920. Figure 4b presents the corresponding training and validation loss across the same epoch range. The training loss starts relatively high at ~0.61 in the 0_*th*_ epoch and shows a steady decline, reaching around 0.13 by the 17_*th*_ epoch. The validation loss follows a more irregular pattern, beginning near 0.40, spiking intermittently, and settling at around 0.33 in the final epoch.

**Figure 5:**
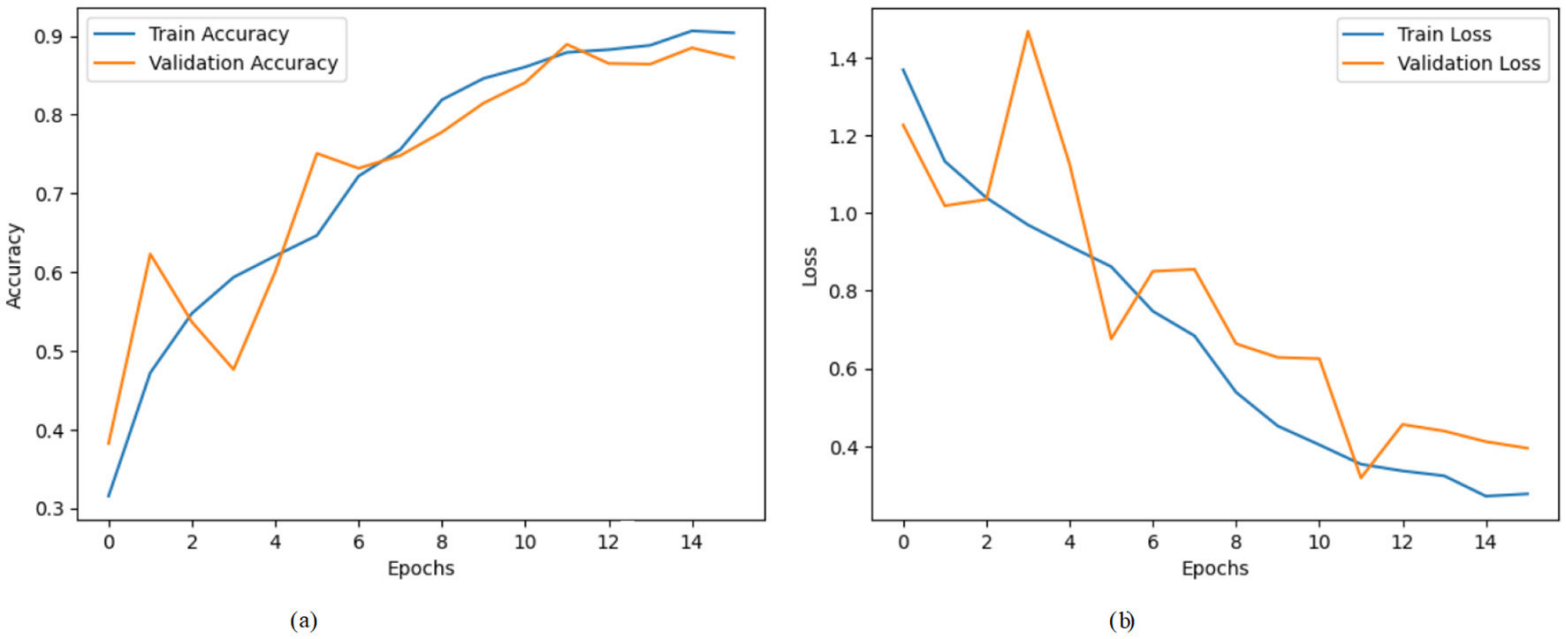
Graphical representation of hybrid CNN-VGG16 model with XAI on third dataset. **(a)** Accuracy graph. **(b)** Loss graph.

Figure 5a shows a model's training and validation accuracy over 15 epochs. The training accuracy starts at ~0.310 at the 0_*th*_ epoch and rises steadily throughout the training process, reaching about 0.905 by the 15_*th*_ epoch. The validation accuracy initially starts higher at around 0.390, increases with some fluctuations, and peaks around 0.890 near the 11_*th*_ epoch before settling slightly lower at ~0.875 by the final epoch. Figure 5b presents the corresponding loss values over the same epoch range. The training loss begins at a relatively high value of around 1.38 at the 0_*th*_ epoch and decreases consistently, dropping to ~0.28 by the 15_*th*_ epoch. The validation loss starts at about 1.22 and fluctuates more than the training loss, reaching a peak around 1.48 at the 3_*rd*_ epoch but then follows a general downward trend to around 0.40 at the final epoch.

Table 1 presents the classification performance across three datasets: Brain Tumor MRI, Augmented Alzheimer MRI, and a third tumor classification dataset. The Brain Tumor MRI dataset includes four tumor classes: glioma_tumor, meningioma_tumor, no_tumor, and pituitary_tumor. The model achieves the highest F1-score of 0.98 for the pituitary_tumor class, with corresponding precision and recall values of 0.97 and 0.99, respectively. The glioma_tumor class also performs strongly with all three metrics: precision, recall, and F1-score at 0.96. The no_tumor class has a slightly lower recall of 0.87, contributing to an F1-score of 0.90. Overall, the model demonstrates high classification effectiveness with a total accuracy of 94%. In the Augmented Alzheimer MRI dataset. This dataset includes four classes: MildDemented, ModerateDemented, NonDemented, and VeryMildDemented. Among these, the NonDemented class achieves the highest F1-score of 0.87, driven by a strong recall of 0.89. Although the ModerateDemented class attains a perfect precision of 1.00, its low recall of 0.54 results in a moderate F1-score of 0.70, indicating potential challenges in correctly identifying all instances of this class. The overall model accuracy for this dataset is 81%, which suggests reasonable but improvable classification performance. The third dataset consists of the following classes: glioma, meningioma, no tumor, and pituitary. The tumor class performs the best with an F1-score of 0.97, bolstered by a precision of 0.96 and a recall of 0.98. The pituitary class also achieves high recall (0.99), although its precision is relatively lower at 0.88, yielding an F1-score of 0.93. The overall model accuracy stands at 93%, indicating a strong performance across multiple tumor categories.

**Table 1:** Classification metrics across three datasets.

**Dataset**	**Class**	**Precision**	**Recall**	**F1-Score**
Brain tumor MRI	Glioma_tumor	0.96	0.96	0.96
	Meningioma_tumor	0.91	0.91	0.91
	No_tumor	0.92	0.87	0.90
	Pituitary_tumor	0.97	0.99	0.98
	**Accuracy**	94%		
Augmented Alzheimer MRI	MildDemented	0.81	0.64	0.72
	ModerateDemented	1.00	0.54	0.70
	NonDemented	0.84	0.89	0.87
	VeryMildDemented	0.76	0.77	0.76
	**Accuracy**	81%		
Third dataset	Glioma	0.96	0.89	0.92
	Meningioma	0.92	0.83	0.87
	Notumor	0.96	0.98	0.97
	Pituitary	0.88	0.99	0.93
	**Accuracy**	93%		

For multi-class classification, SHAP values were calculated per class and reshaped for visualization. Summary plots were generated to identify globally important regions across all samples.

### 4.1 Model explainability using SHAP

To better understand how our Hybrid CNN + VGG16 model makes decisions, we used SHapley Additive explanations (SHAP). This method explains model predictions by highlighting which parts of the input image contribute most to the final output. Since our model has a dual-input architecture with the same MRI image passing through two branches for enhanced feature learning, we adapted SHAP's DeepExplainer to handle this structure accordingly. We selected a sample batch from the validation set and computed SHAP values for both inputs. Summary plots were generated to identify which features (or pixel regions) are typically important over the dataset and image plots for each pixel that mattered in discriminating a given prediction from the others. This allowed these visualizations to show that no matter the input, the model always attends to brain regions involved in Alzheimer's disease. This provides valuable guidance for building trust in AI-based clinical tools, and the model is strengthened in terms of interpretability and communicates that it is learning meaningful patterns.

In order to increase the interpretability of the hybrid CNN+ VGG16 model trained for the brain tumor classification task, we combined the SHapley Additive exPlanations (SHAP) technique that allows explainable AI. The model has a multi-input structure (perhaps there is a better term for this), so SHAP's DeepExplainer was used on batches of validation images to compute pixel-based contributions for each prediction. The SHAP values revealed which areas of the MRI scans were the most important in allowing the model to decide. We would find through summary plots that the model consistently locked in on key tumor areas irrespective of the different categories, thus showing that it accurately emphasized those features. However, this transparency not only supports the credibility of the model but, additionally, is of the essence for the reliability of AI-based diagnostics in other medical applications.

#### 4.1.1 SHAP summary plots of second dataset

SHAP values were successfully computed for a multi-input model using DeepExplainer, with each input consisting of 32 RGB images (128 × 128). The resulting SHAP tensors had a shape of (32, 128, 128, 3, 4), indicating class-specific attributions. Separate summary plots were generated for the four classes across both inputs, highlighting important spatial regions contributing to the model's predictions.

**Figure 6:**
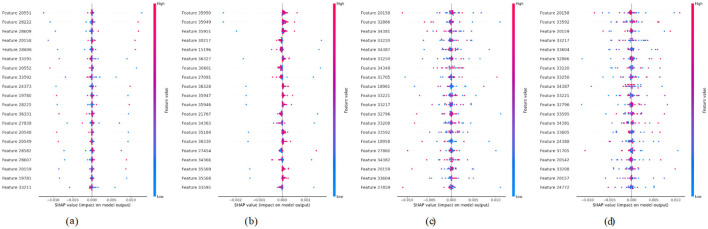
SHAP summary plots for Classes 0 through 3. **(a)** Class 0. **(b)** Class 1. **(c)** Class 2. **(d)** Class 3.

Figure 6a presents a SHAP summary plot that visualizes the influence of Features labeled numerically from 20551 to 36331. The *x*-axis represents SHAP values, where positive values indicate features that push the prediction higher, and negative values indicate the opposite. Color gradients reflect feature magnitudes. Pink denotes high values, and blue denotes low values. In this case, certain features like 20551 and 28222 exhibit a more pronounced impact on the model's predictions, evidenced by their wider spread along the SHAP value axis compared to others. On the other hand, features such as 20548 and 20549 show minimal impact, clustering closer to zero. Figure 6b presents a SHAP summary plot that illustrates the influence of features from “Feature 35950” to “Feature 33595” on the model's output. Notably, 35950 and 35184 are significantly influenced by their pronounced spread along the SHAP value axis, suggesting they contribute meaningfully to the model's output. In contrast, features like 21767 and 35569 cluster closer to zero, indicating a minimal effect on the predictive performance. Figure 6c presents a SHAP summary plot that illustrates the features that influence the model's output, ranging from “Feature 20158” to “Feature 27859.” Notably, features such as “Feature 20158” and “Feature 34381” significantly impact the model's predictions, as indicated by the broader distribution of SHAP values. This suggests that variations in these features can lead to more pronounced effects on the predictions. In contrast, features like “Feature 34348” and “Feature 18958” cluster closer to the zero line, indicating a lesser impact on model predictions. This clustering reveals that changes in these features do not significantly influence the overall model output. Figure 6d presents a SHAP summary plot that visualizes the influence of features ranging from “Feature 20158” to “Feature 24772” on the model's output. For instance, Features 20158 and 33604 exhibit strong positive contributions when their values are high, whereas Features 33250 and 24772 predominantly display negative SHAP values, indicating a suppressive effect on predictions. This plot highlights key features that significantly shape model behavior based on their value ranges.

**Figure 7:**
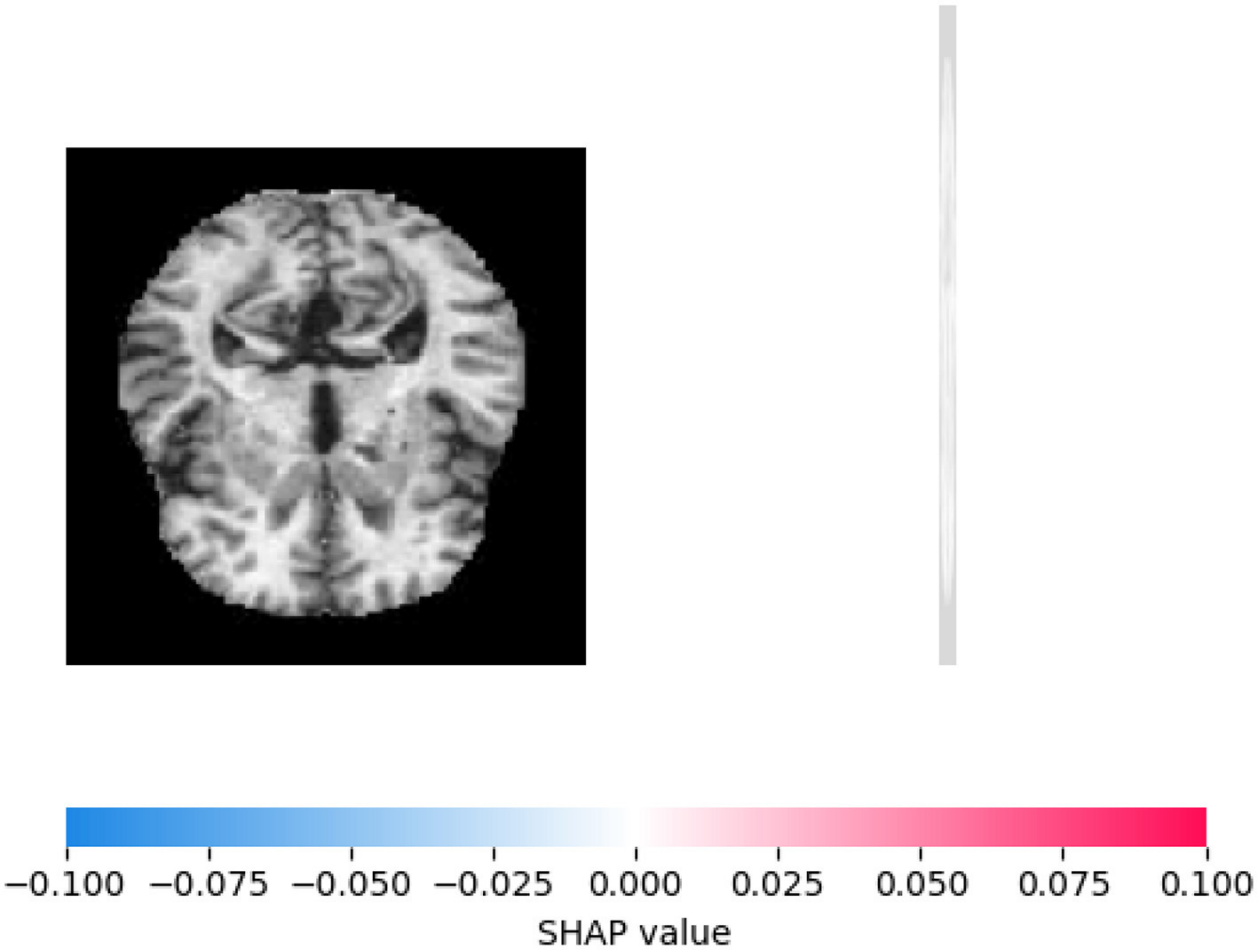
Visualizing SHAP explanation for sample 0, class 0.

Figure 7 displays a cross-sectional brain image alongside a SHAP value color scale. The grayscale brain scan highlights structural features, while the adjacent gradient from blue (–0.1, negative contribution) to red (+0.1, positive contribution) represents each region's influence on model predictions. This integration aids in interpreting how specific brain areas affect analytical outcomes, linking neuroimaging data to model behavior.

#### 4.1.2 SHAP summary plots of third dataset

**Figure 8:**
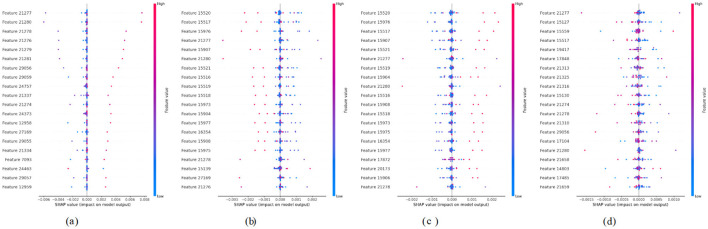
SHAP summary plots for Classes 0 to 3. **(a)** Class 0. **(b)** Class 1. **(c)** Class 2. **(d)** Class 3.

Figure 8a presents a SHAP summary plot that illustrates the impact of various features, ranging from “Feature 21277” to “Feature 12959,” on the model's predictions. The visualization indicates that certain features, such as “Feature 21280” and “Feature 29056,” significantly influence the model's output, as evidenced by their extensive spread along the SHAP value axis. In contrast, features like “Feature 21337” and “Feature 24373” demonstrate minimal impact, as their SHAP values cluster closer to zero. Figure 8b presents a SHAP summary plot visualizing the influence of various features, specifically labeled from “Feature 15520” to “Feature 21276,” on the model's output. In this plot, features such as “Feature 15976” and “Feature 15908” exhibit a significant influence, as indicated by their wider dispersion on the SHAP value axis. This means that these features contribute more substantially to the predicted outcomes when compared to others. Conversely, features like “Feature 15520” and “Feature 15139” cluster closer to zero, demonstrating minimal impact on the model's predictions. Figure 8c presents a SHAP summary plot that illustrates the influence of various features, specifically from “Feature 15520” to “Feature 21278,” on the model's predictions. Certain features, such as “Feature 15520” and “Feature 15976,” exhibit a more pronounced effect on the model's predictions, as evidenced by their greater dispersion along the SHAP value axis. This suggests that these features are critical in influencing the model's output. Conversely, features like “Feature 15518” and “Feature 15904” reveal a minimal impact, clustering closely to zero. This suggests that their contributions to the model's predictions are negligible compared to those of other features. Figure 8d presents a SHAP summary plot that represents the impact of various features on the model's predictions, focusing on features ranging from “Feature 21277” to “Feature 21659.” For instance, features such as “Feature 21280” and “Feature 29056” significantly impact the predictions, as indicated by their wider distribution of SHAP values that extend toward both positive and negative extremes. Conversely, features like “Feature 17104” and “Feature 15130” exhibit minimal influence, clustering closer to the zero mark, which suggests that their effect on the model output is negligible.

**Figure 9:**
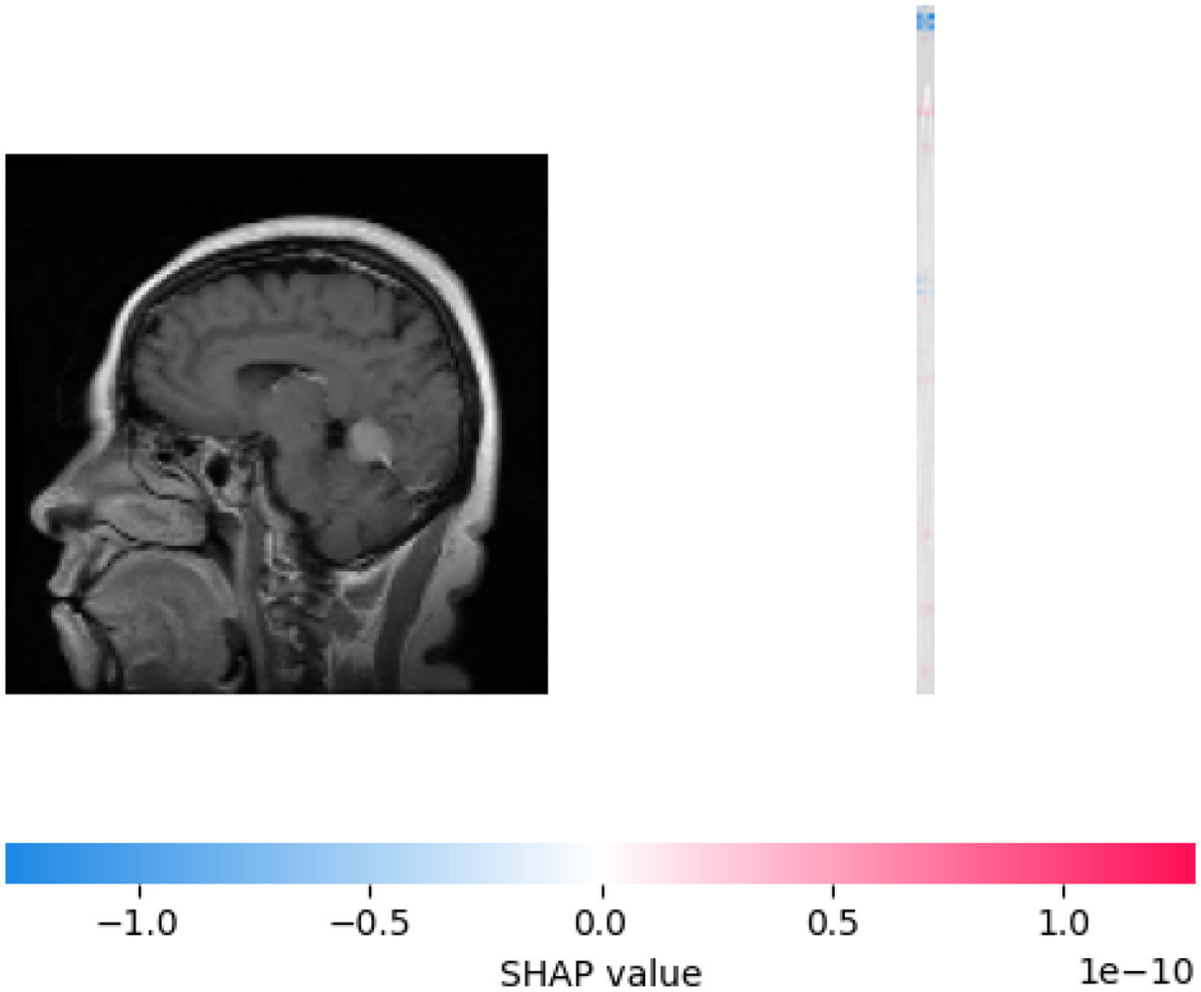
Visualizing SHAP explanation for sample 0, class 0.

Figure 9 combines a sagittal brain MRI image (left) with a SHAP value bar plot (right) to illustrate model interpretability in neuroimaging. The MRI highlights anatomical brain structures, while the SHAP plot uses a blue-to-red gradient to show each region's contribution to model predictions, with blue indicating a negative and red indicating a positive influence. SHAP values range from –1 to 1, capturing features' subtle and significant impacts. This integrated visualization aids in understanding how specific brain regions affect model outcomes, bridging neuroimaging with explainable AI.

### 4.2 Discussion

The proposed hybrid CNN-VGG16 framework addresses three key challenges in MRI-based neuroimaging diagnostics: limited labeled data, variability across datasets, and lack of interpretability in deep learning models. First, the use of transfer learning significantly mitigates the issue of data scarcity. By leveraging the pre-trained VGG16 architecture, the model benefits from rich feature representations learned from large-scale natural image datasets. This allows for effective feature extraction even with relatively small medical imaging datasets. The high classification accuracy achieved on the brain tumor dataset (94%) and the third dataset (93%) demonstrates the model's ability to generalize across similar pathological domains. Second, the sequential fine-tuning strategy across structurally distinct datasets of brain tumors and Alzheimer's and a third validation set demonstrates the framework's adaptability to different neuroimaging modalities. The model maintains a competitive performance of 81% on the augmented Alzheimer dataset despite its structural differences from the training domain. This highlights the framework's robustness and transferability, addressing the domain shift problem that often limits the practical deployment of deep learning models in medical diagnostics. Third, integrating SHAP-based Explainable AI resolves the critical issue of interpretability. By generating pixel-level explanations, the framework provides insight into which brain regions influence the model's predictions. This capability enhances clinical trust and offers potential support for diagnostic reasoning by aligning model attention with known anatomical and pathological patterns. The proposed approach combines performance and transparency, offering a concrete step toward clinically viable AI systems. It outperforms traditional single-dataset training and black-box models by effectively resolving challenges related to data diversity, cross-domain generalization, and explainability.

## 5 Conclusion

This paper demonstrated the effectiveness of transfer learning combined with XAI for classifying MRI images. SHAP values provide much insight into the decision-making path of the model, and the hybrid CNN-VGG16 model generalizes well over different datasets with high accuracy. In conclusion, this approach and its generalizations can be applied to other medical imaging tasks, possessing high performance and interpretability. This research has demonstrated the effectiveness of a hybrid CNN-VGG16 model, utilizing transfer learning in conjunction with XAI techniques, for MRI image classification. The high accuracy of the model across multiple datasets demonstrates that it is robust and easily adaptable in distinguishing between different neurological diseases, including brain tumors and Alzheimer's disease. While the model shows strong performance, it has certain limitations. The reliance on a limited number of public datasets may restrict its generalizability to real-world clinical scenarios. Additionally, the SHAP-based interpretability comes with a high computational cost, which may challenge real-time deployment. Future work will expand dataset diversity, incorporate 3D volumetric data, optimize model architecture for clinical deployment, and explore alternative interpretability methods. This research lays a solid foundation for developing high-performing, interpretable AI tools to support medical decision-making and improve patient outcomes. This work also lays the groundwork for future research to refine the model further and apply it to other medical imaging applications, ultimately leading to enhanced patient outcomes.

## Data Availability

The original contributions presented in the study are included in the article/supplementary material, further inquiries can be directed to the corresponding authors.
